# Vernal growth of vocal control nucleus Area X, but not HVC, precedes gonadal recrudescence in wild black‐capped chickadees (*Poecile atricapillus*)

**DOI:** 10.1111/jne.13375

**Published:** 2024-02-20

**Authors:** Broderick M. B. Parks, Kyle McVea, Leslie S. Phillmore

**Affiliations:** ^1^ Department of Psychology and Neuroscience Dalhousie University Halifax Nova Scotia Canada

**Keywords:** black‐capped chickadees, hypothalamic–pituitary–gonadal axis, neural plasticity, seasonality, vocal control system

## Abstract

In temperate‐zone songbirds, the neuroanatomical changes which occur in advance of breeding, including the growth of nuclei of the vocal control system, are believed to occur downstream of gonadal recrudescence. However, evidence from wild birds is mixed. Here, we captured black‐capped chickadees from the wild in early spring (March–April), summer (August–September), and winter (December–January); in addition to measuring the volumes of two vocal control nuclei (Area X and HVC), we also quantified two indicators of reproductive state (gonads and circulating gonadal steroids). Most birds captured in early spring had regressed gonads and low levels of circulating gonadal steroids, indicating these birds were not yet in full breeding condition. However, these early spring birds still had a significantly larger Area X than winter birds, while HVC did not differ in size across groups. Using data from a previously published seasonal study of black‐capped chickadees (Phillmore et al., *Developmental Neurobiology,* 2015;75:203–216), we then compared Area X and HVC volumes from our early spring group to a breeding group of chickadees captured 3–4 weeks later in the spring. While Area X volume did not differ between the studies, breeding males in Phillmore et al. (2015) had a significantly larger HVC. Taken together, this suggests that the vernal growth of Area X occurs ahead of HVC in black‐capped chickadees, and that the overall vernal changes in the vocal control system occur at least partially in advance of the breeding‐associated upregulation of the hypothalamic–pituitary–gonadal axis.

## Graphical abstract

According to the longstanding model of vernal recrudescence of the songbird vocal control system, upregulation of the hypothalamic‐pituitary‐gonadal axis, and specifically testosterone (T), promotes growth first of HVC, and then Area X. In this study, we show that in black‐capped chickadees, Area X is already fully recrudesced ahead of HPG axis upregulation and in advance of HVC, suggesting that HVC growth is not required to effectuate Area X growth. We also show that in males, HVC is significantly larger in the late spring (when gonads are large) compared to the early spring (when gonads are not fully recrudesced), suggesting the growth of HVC, but not Area X, may be regulated by HPG axis upregulation.

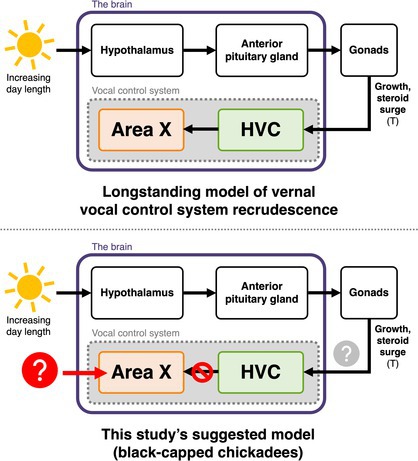

## 1 INTRODUCTION

In temperate‐zone songbirds, the transition from non‐breeding to breeding state is accompanied by a suite of physiological changes, including an upregulation in activity of the hypothalamic–pituitary–gonadal (HPG) axis.^1^, ^2^ Downstream effects of HPG axis upregulation include gonadal recrudescence and a subsequent surge in circulating gonadal steroids.^3^, ^4^ Behaviorally, many male songbirds exhibit a breeding‐associated increase in song production (also females of some species^5^, ^6^), generally for the purposes of mate attraction.^7^, ^8^ Song output often increases in breeding season relative to non‐breeding season^9^, ^10^; manipulating reproductive state in laboratory‐housed songbirds via photoperiod or exogenous steroid administration can likewise modulate song production.^11^ In many songbirds, the neural structures supporting song and other vocalizations, known collectively as the vocal control system, show evidence of seasonal plasticity in tandem with seasonal plasticity in song production^12^, ^13^, ^14^: Area X, HVC (letter‐based proper name), and the robust nucleus of the arcopallium (RA), among others, tend to be larger in breeding birds compared with non‐breeding birds. Other morphological and connective attributes within these nuclei (e.g., neuron number, soma size, neurogenesis) also change seasonally in many species.^14^, ^15^

The relationship between timing of activation of the HPG axis and growth in the vocal control system has not been fully elucidated. Some volumetric changes in the vocal control system are regulated in part by gonadal steroids, including testosterone (T),^16^ leading to the hypothesis that the vernal growth of the vocal control system is a direct downstream result of vernal HPG axis upregulation, particularly the surge in T (e.g., Refs. [8, 17, 18, 19, 20]). However, empirical evidence from wild birds supporting this longstanding model varies. For example, wild song sparrows (*Melospiza melodia*) captured in late winter (February) had significantly larger HVC and RA by volume compared with birds captured in the late fall (October–December), despite non‐breeding levels of circulating T.^21^ Similarly, in male blue tits (*Cyanistes caeruleus*) both HVC and RA are fully developed in February, well in advance of peak HPG axis activity.^22^ This suggests that vocal control system growth is not delayed until T is at maximal levels, but is instead at least initiated in advance of reproductive maturation, perhaps as T levels are rising in response to increasing photoperiod.

While laboratory manipulations of reproductive state necessitate at least some form of captivity, captivity itself may also modulate changes in the HPG axis. Birds photostimulated in captivity tend to have lower levels of circulating androgens than their wild counterparts.^23^, ^24^ Captivity also appears to constrain changes in volume of vocal control nuclei.^23^ Perhaps no better example of such discrepancies is seen in studies of black‐capped chickadees (*Poecile atricapillus*), a non‐migratory North American parid studied extensively in the context of seasonal changes in spatial behavior and the hippocampus.^25^, ^26^ These discrepancies also extend to the chickadee vocal control system. For example, while MacDougall‐Shackleton et al.^27^ showed robust volumetric differences in Area X, HVC, and RA in chickadees housed on different photoperiods in extended captivity (4–6 months), no subsequent study has fully replicated these findings. In contrast, Smulders et al.^28^ found no seasonal differences in any vocal control structure in a wild‐caught population of chickadees captured over the course of the year and not introduced to captivity. Birds housed for intermediate periods of captivity appear to have similarly intermediate seasonal differences in vocal control system region volumes: HVC, but not Area X, is larger in birds categorized as breeding compared with non‐breeding (7 days captivity^29^; overnight captivity^30^).

Here, we examined seasonal changes in the volumes of vocal control nuclei Area X and HVC in a wild population of black‐capped chickadees not exposed to any period of captivity. To assess HPG axis activity, we, as in Phillmore et al.,^29^, ^30^ quantified seasonal changes in gonads (testis volume in males; ovary development in females), and we extend their findings by also quantifying circulating gonadal steroids (T in males; 17β‐estradiol, E2, in females). As birds collected for this study and the birds collected in Phillmore et al.^30^, ^31^ were collected using similar methods and from the same geographical region, but at different times within the breeding season (March–April vs. April–May), we also took the opportunity to directly compare brain data between the two samples of birds to probe for finer‐scale within‐season differences and to assess the timing of volume change in the vocal control system.

## 2 METHODS

All experimental procedures were approved by the Dalhousie University Committee on Laboratory Animals (permit no. 21‐021) in accordance with the guidelines of the Canadian Council on Animal Care. Birds were captured and handled under a Scientific Permit (no. SC4055) issued by the Canadian Wildlife Service (CWS), Environment and Climate Change Canada.

### 2.1 Animals and capture seasons

We captured black‐capped chickadees (*N =* 48) from private field sites in Halifax, Nova Scotia, Canada across three seasons: a spring group (*n* = 16) between 25 March – 10 April 2021, a summer group (*n* = 16) between 13 August and 15 September 2021, and a winter group (*n* = 16) between 1 December 2021 and 26 January 2022. We made a priori estimations of photoperiodic condition (i.e., spring = photostimulated; summer = photorefractory; winter = photosensitive) based on local day length, as well as timelines used in previous seasonal studies of black‐capped chickadees in the area,^30^, ^31^ however in this study, we captured the spring group earlier than the breeding birds in Phillmore et al.^30^, ^31^ according to the parameters of our CWS Scientific Permit.

### 2.2 Capture procedure

Birds were captured using walk‐in (Potter) traps baited with black oil sunflower seeds between the hours of 08:30 and 11:30, at least 60 min after the local sunrise time to avoid the influence of diel variation in hormones. To attract birds to the traps, we played audio recordings of black‐capped chickadee mobbing calls from a nearby speaker; recordings were obtained with permission from the MacAulay Library at the Cornell Lab of Ornithology (ML14646, ML205639, and ML195523) and xeno‐canto (XC544961). Upon capture, we stopped playback and immediately extracted the bird, estimated age and sex (described below) and then collected whole blood via brachial venipuncture into 70 μL heparinized microcapillary tubes (maximum 140 μL per bird; cat no. 22‐363‐566, Fisherbrand). Tubes were then sealed with clay (Critoseal, McCormick Scientific) and stored on ice until processing. Birds were then secured in a cloth bird bag and transported via vehicle to the laboratory. As we wanted to avoid potential captivity effects, we were mindful of minimizing the amount of time between capture and sacrifice: once the first captured bird was secured in the bird bag, we generally reset traps and restarted playback to capture additional birds for a maximum of about 30 min, after which all birds captured and bled were transported to the laboratory by vehicle (mean time between capture and departure from field site = 21 min, range = 7–39 min).

### 2.3 Aging and sexing procedure

An overview of the number of birds for which there is data (split by season, sex, and age) per dependent measure is presented in Table 1. As we aimed to have an equal number of males and females in each condition, there were several instances where birds were measured (for wing chord, tail length) before blood collection, but released if the bird did not balance the group (i.e., for sex) or appeared too young. However, given the degree of overlap in external morphological characteristics between males and females,^32^ sex was not confirmed until examination of gonads (described below) and therefore sample sizes across conditions are not exactly equal. Similarly, although we aimed to include only adults (hereafter after‐hatch‐year, AHY^32^), we ultimately kept several juvenile (hereafter hatch‐year, HY) birds, particularly in the summer group. Black‐capped chickadees are difficult to age in the field,^32^ especially when there are time constraints associated with capture and handling, and because the field marks use to distinguish AHY and HY chickadees (e.g., wear, color of outer rectrices, color of upper mandible lining^32^) are fairly unreliable.^33^ Thus age was not confirmed until a post‐perfusion examination of skull pneumatization, a more robust and reliable indicator of age in songbirds,^32^ including black‐capped chickadees.^34^, ^35^ We therefore examined age differences in the summer group only (described separately below); one HY bird captured in winter was excluded from analyses. All other seasonal analyses were conducted using AHY birds only.

**TABLE 1:** Group sample sizes for all dependent measures, split by season, sex, and age.

Measure		AHY			HY
		(*n* = 36)			(*n* = 11)
		Spring	Summer	Winter	Summer
Testis volume (♂ only)		8	2	7	8
Ovary score (♀ only)		8	3	7	3
Plasma T (♂ only)		5	1	3	7
Plasma E2 (♀ only)		4	2	2	1
Area X volume	♂	6	2	8	8
	♀	8	3	7	3
HVC volume	♂	7	2	8	7
	♀	7	2	6	2

*Note*: AHY, after‐hatch‐year; HY, hatch‐year; T, testosterone; E2, 17β‐estradiol. AHY birds captured in summer were included in both seasonal analyses (with other AHY birds) and exploratory age analyses (with HY birds). Although we captured 48 birds, we excluded the one HY bird captured in winter (total *N* used in all analyses = 47).

### 2.4 Euthanasia and perfusion

Upon arrival at the laboratory, birds were removed from the bird bag and euthanized with a 0.1 mL intraperitoneal Euthanyl injection. The mean time between capture in the field and euthanasia was 64 min (range = 31–96 min). After euthanasia was confirmed, we exposed and severed the jugular vein and collected trunk blood into heparinized microcapillary tubes (maximum 350 μL per bird). Birds were then transcardially perfused with heparinized phosphate‐buffered saline (PBS; pH = 7.4) followed by 4% buffered paraformaldehyde (PFA; pH = 8.5). The brain was then quickly extracted and submerged in 4% buffered PFA for 24–72 h (until uniformly fixed), then in 30% buffered sucrose for 24–48 h (until saturated), then snap frozen on crushed dry ice and stored at −80°C until sectioning.

We confirmed sex by a post‐mortem examination of the gonads. In males, the left testis was removed; testis length and width were measured (to the nearest 0.1 mm) using dial calipers. These measurements were then used to calculate testis volume using the formula of an ellipsoid:

$$\frac{4}{3}\pi a^{2}b$$

where *a* = width/2 and *b* = length/2. Males with testes larger than 20 mm^3^ are considered to be in breeding condition (i.e., photostimulated^29^). In females, we visually scored the stage of ovary development using a 5‐point ordinal scale originally described for black‐capped chickadees by MacDougall‐Shackleton et al.^36^ Here, we considered females with an ovary score of 3 (small uniform follicles), 4 (hierarchical follicles), or 5 (large yolky follicles) as having “stimulated” ovaries reflective of breeding condition.

### 2.5 Blood processing and hormone assays

Blood samples were processed on the day of capture, at most within 4 h of collection. All tubes containing blood collected in the field and most tubes containing trunk blood were spun on a microhematocrit centrifuge (Unico C‐MH30) at 16,000*g* for 10 min. Plasma was then separated from the blood pellet, aliquoted, and stored at −20°C until processing. Hormone assays were completed only after all samples from all birds were collected for this study. We quantified gonadal steroids (T in males; E2 in females) in plasma using commercially available enzyme‐linked immunosorbent assay (ELISA) kits (T: Enzo Life Sciences, cat no. ADI‐900‐065; E2: Salimetrics, cat no. 1‐3702), following manufacturer instructions. Additional details on hormone assays can be found in Supplemental Materials S1.

### 2.6 Tissue sectioning

Before sectioning, a brain was removed from the −80°C freezer and placed in a cryostat (Leica CM1950); once warmed to −16°C, we separated the hemispheres using a razor blade. One hemisphere was then returned to −80°C and the other was mounted on the cryostat and sectioned coronally at 30 μm into four sets. We alternated which hemisphere was sectioned and which was returned to the freezer based on order of capture; the hemisphere to be sectioned from the first bird was chosen at random. Once sectioned, tissue was stored in cryopreservative (30% sucrose, 30% ethylene glycol, 1% polyvinylpyrrolidone in PBS) at −20°C until processing. All brains were sectioned only after all birds were collected for this study. At the time of sectioning, birds were assigned a random three‐digit identification number such that all subsequent tissue processing, microscopy, and volume reconstruction could be completed blind to season, sex, and age.

### 2.7 Brain region morphometry

**FIGURE 1:**
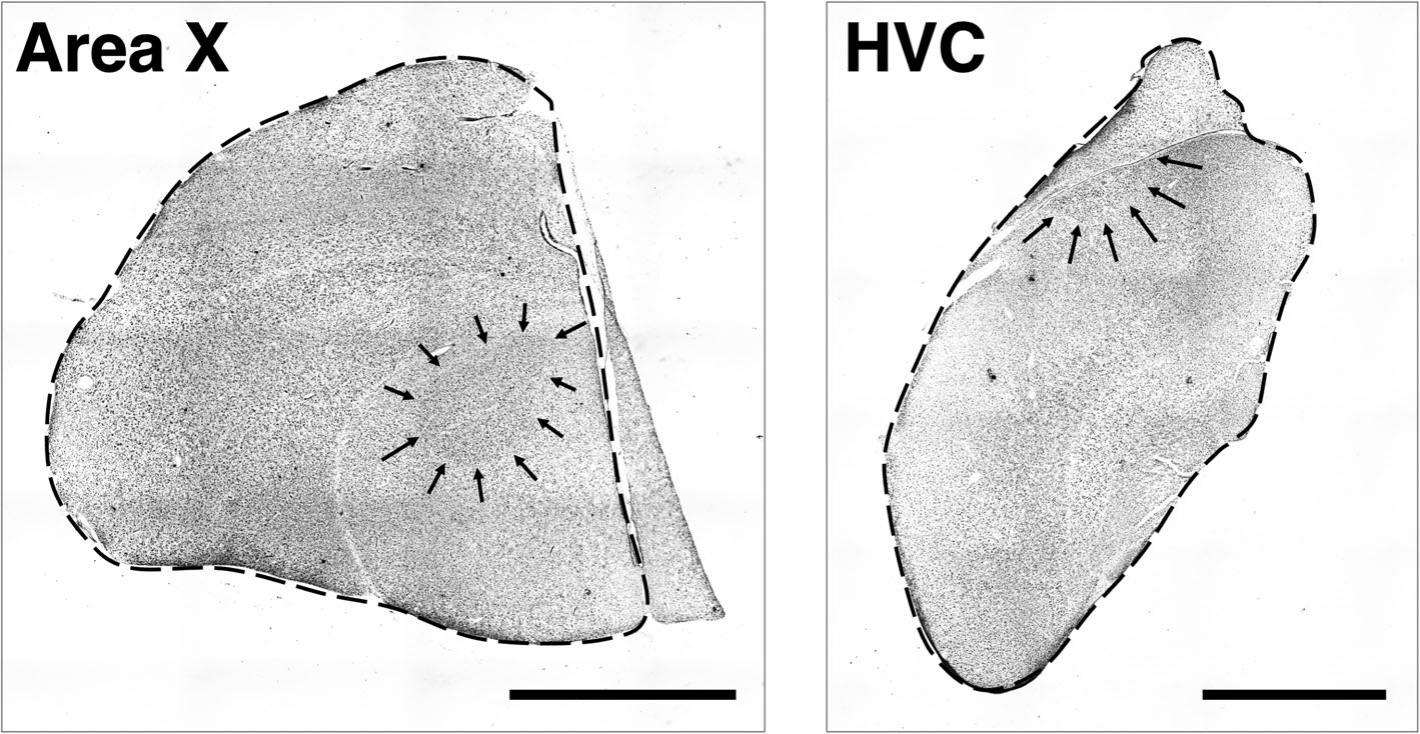
Photomicrographs of black‐capped chickadee coronal brain sections (one hemisphere) stained with cresyl violet; arrows indicate boundaries of vocal control nuclei Area X (left) and HVC (right). Dorsal boundary of HVC is the lateral ventricle (not indicated). Dashed line indicates telencephalon. Scale bars (bottom right) indicate 2 mm.

We stained one set of tissue for Nissl substance using cresyl violet (for additional details on protocol, see Supplemental Materials S1). Cresyl violet‐stained sections were imaged under brightfield illumination using an Olympus DP80 camera paired to an Olympus BX‐51 microscope with an automated motorized stage (Prior ProScan II). We used the Multiple Image Alignment feature in Olympus cellSens Dimension software (version 1.14) to scan and capture individual images (using a ×4 objective; 680 × 512 resolution, 309.5 pixels/mm), stitching them into whole‐slide images. Whole‐slide images were then imported into the FIJI version of ImageJ (version 1.53q^37^), where we used the Polygon function to trace the area of Area X, HVC, and the telencephalon (Figure 1). We traced the area of both Area X and HVC in every section in which they were visible (sampling interval = 120 μm) using a stereotaxic atlas of the zebra finch (*Taeniopygia castanotis*) brain^38^ as reference. We then calculated the volume (*V*) between each section using the formula for a frustum:

$$V=\frac{W}{3\left(A_{1}+\left(\sqrt{A_{1}}\times \sqrt{A_{2}}\right)+A_{2}\right)}$$

where *A*_1_ and *A*_2_ are areas from successive sections and *W* is the interval between the sections. We calculated total nucleus volume by summing the volumes of each frustum. We used the same method to calculate telencephalon volume, except we sampled from every second mounted section (sampling interval = 240 μm). When measuring the volume of the vocal control nuclei and the telencephalon, we were generally able to account for any sections that were damaged or missing by adjusting the sampling interval (*W*). However, volume measurements for Area X and HVC were excluded from 2 and 4 birds respectively, due to issues with tissue quality and/or poor staining (Table 1). Volume measurements for all structures (Area X, HVC, telencephalon) were both highly repeatable within observers (intraclass correlation coefficients = .967–.999, all *p*s < .001) and consistent between observers (ICCs = .818–.991, all *p*s < .01; additional information in Supplemental Materials and Table S1).

### 2.8 Comparison with Phillmore et al.^30^

The birds used for the studies by Phillmore et al.^30^, ^31^ were captured in two groups (breeding/non‐breeding) from the same geographical region (Halifax, NS) over a decade ago (2008–2009). Those birds were housed in captivity overnight and sacrificed the following day, and a subset of brain tissue was used to reconstruct the volumes of Area X and HVC according to the same method as the present study. Gonad data were published in Phillmore et al.^31^; vocal control volumes were published in Phillmore et al.^30^

We were specifically interested in comparing physiological and neuroanatomical data between the spring males in the present study (captured between 25 March and 10 April 2021) and the breeding males in Phillmore et al.,^30^ given the 3–4 week difference in capture dates. Phillmore et al.^30^ quantified Area X and HVC volume in both the left and right hemisphere for each bird; due to the design of the present study we could not examine hemispheric differences so we compared the absolute volumes (one hemisphere) of birds in the present study to the average absolute volume (by averaging across left and right hemisphere) of the birds in Phillmore et al.^30^ We only compared data from males, as tissue collected from females in Phillmore et al.^30^ was both processed and analyzed differently from the birds in the present study, and also included a subset of females captured in a different geographic location.

### 2.9 Statistical analyses

All analyses were conducted using jamovi (version 2.3.21^39^), an open‐access statistical programming software integrated with R (version 4.1^40^). We used different models based on the type of data being analyzed (described below); data were screened for normality and homogeneity of variances before analyses (using Shapiro–Wilk and Levene's tests, respectively) and transformed where necessary. We did not exclude any outliers from analyses. Unless otherwise indicated, data were analyzed with linear models using the GAMLj module (General Analyses for Linear Models in jamovi; version 2.6.6^41^); all post hoc tests of main effects consisted of multiple pairwise comparisons and all *p*‐values derived from multiple pairwise comparisons were adjusted using the Bonferroni correction (denoted as *p*_B_). Significance was set at *ɑ* = .05.

## 3 RESULTS

### 3.1 Adults (HY birds)

#### 3.1.1 Physiological measures

**FIGURE 2:**
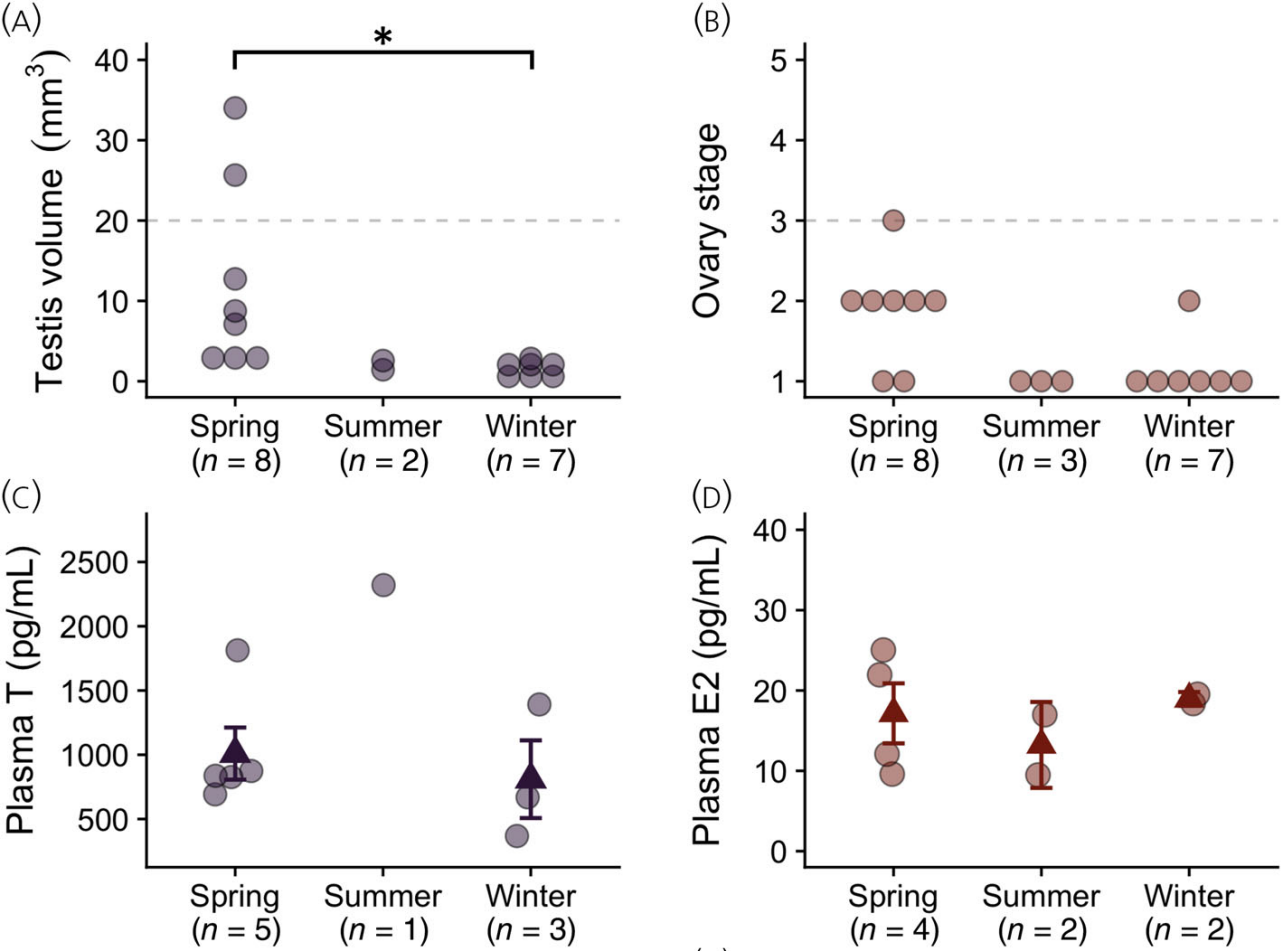
Seasonal differences in HPG axis activity in adult (AHY) black‐capped chickadees. (A) shows testis volume in males; dashed line indicates 20 mm^3^ threshold for breeding condition described by Phillmore et al.^29^ Asterisk (*) indicates *p* < .05 when analyzing natural‐log transformed testis volume (not presented here for ease of visualization). (B) shows stage of ovary development in females; dashed line indicates stage ≥3 threshold for breeding condition. (C) shows plasma testosterone (T) in males; and (D) shows plasma 17β‐estradiol (E2) in females. Dots are individual data; darker triangles ± error bars (C,D) indicate group means ± SEs.

As shown in Figure 2A, only 2 of the 8 males captured in spring met the threshold for breeding condition described by Phillmore et al.^29^; all other spring males had testes smaller than 20 mm^3^ (mean testis volume ± SE for all spring males = 12.11 ± 4.13 mm^3^). All males captured in the summer and winter had regressed testes smaller than 20 mm^3^. We tested for seasonal differences in testis volume using a general linear model (GLM); testis volume (natural‐log transformed) was the dependent variable and season was the fixed factor. We found a main effect of season on testis volume (*F*_2,14_ = 8.08, *p* = .005; *ω* ^2^ = .455); post hoc tests revealed testes were significantly larger in spring males than in winter males (*t*_14_ = 3.95, *p*_B_ = .004), but not summer males (*t*_14_ = 1.92, *p*_B_ = .227), and did not differ between summer and winter males (*t*_14_ = 0.66, *p*_B_ = 1.00).

In females, only one bird (captured in spring) had stimulated ovaries; all other females had ovary scores of 1 or 2 (Figure 2B). Due to the ordinal nature of the scale used to assess ovary development in female black‐capped chickadees, we analyzed ovary data using a non‐parametric Kruskal–Wallis ANOVA (e.g., Refs. [42]); ovary score was the dependent variable and season was the grouping variable. While we did find a main effect of season on ovary score ($\chi_{2}^{2}=7.67$, *p* = .022), probing the effect further using Dwass–Steel–Critchow–Fligner pairwise comparisons did not yield any significant differences (all *p*s > .05).

We tested for seasonal differences in circulating gonadal steroids using GLMs; hormone concentration was the dependent variable and season was the fixed factor. In males, we found no main effect of season on plasma T (*F*_2,6_ = 3.88, *p* = .083, *ω* ^2^ = .390; Figure 2C). Excluding the one summer male and only comparing spring and winter males also yielded no significant differences (Student's *t*_6_ = 0.571, *p* = .589). In females, there was similarly no main effect of season on plasma E2 (*F*_2,5_ = 0.446, *p* = .664, *ω* ^2^ < 0; Figure 2D).

#### 3.1.2 Neural measures

**FIGURE 3:**
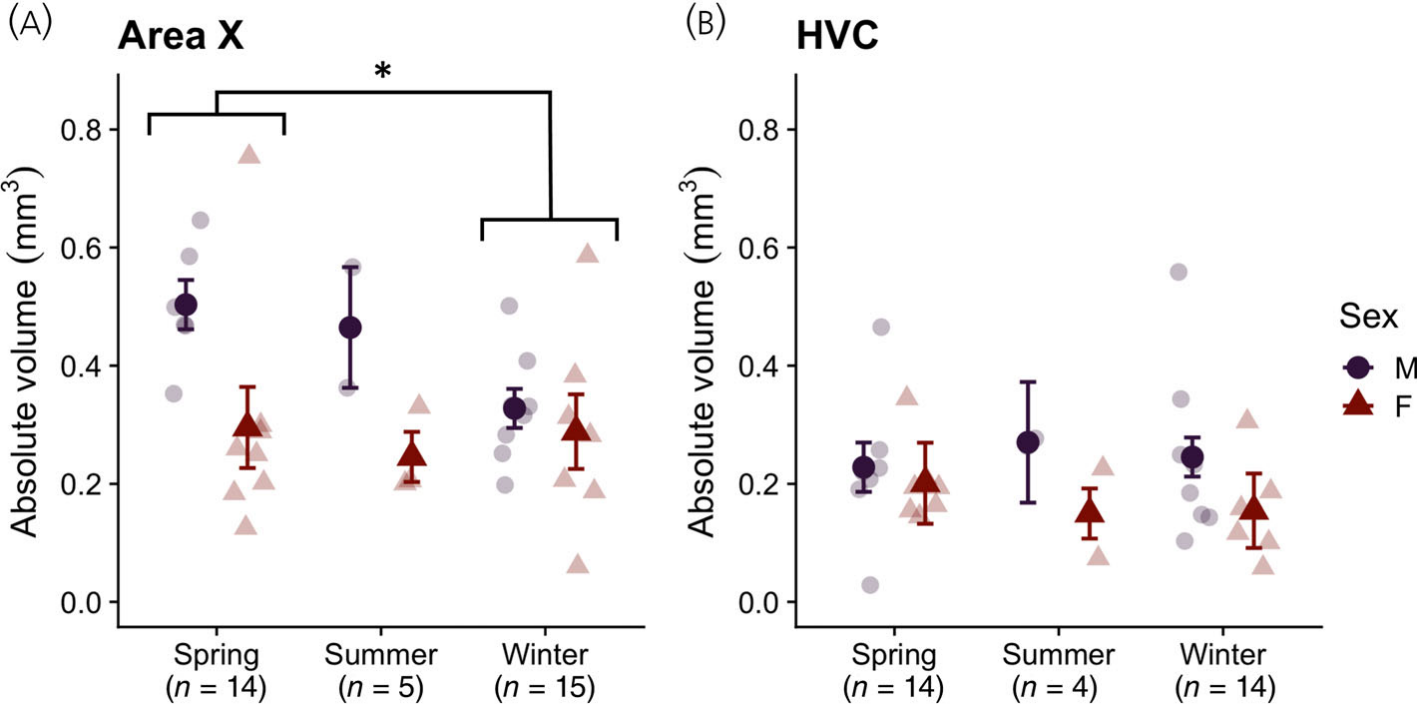
Seasonal differences in vocal control nuclei Area X (A) and HVC (B) in adult (AHY) black‐capped chickadees, separated by sex. Asterisk (*) indicates significant difference at *p* < .05. Faded points are individual data; darker points ± error bars indicate group means ± SEs.

Volume data were analyzed using GLMs. We examined each brain region separately. In each GLM, volume (non‐transformed) was the dependent variable and fixed factors were season and sex. We controlled for individual variation in nucleus volume by including telencephalon volume as a covariate (e.g., Refs. [28, 43, 44]). In Area X (Figure 3A), there was a main effect of sex (*F*_1,27_ = 7.02, *p* = .013, *ω* ^2^ = .08) and season (*F*_2,27_ = 3.46, *p* = .046, *ω* ^2^ = .07), but no interaction (*F*_2,27_ = 0.90, *p* = .417, *ω* ^2^ < 0). Post hoc tests revealed that overall, males had a significantly larger Area X compared with females (*t*_27_ = 2.65, *p*_B_ = .013), and birds captured in spring had a significantly larger Area X compared with birds captured in the winter (*t*_27_ = 2.63, *p*_B_ = 0.042). Area X volume did not differ between spring and summer birds (*t*_27_ = 1.06, *p*_B_ = .891), nor between summer and winter birds (*t*_27_ = 0.80, *p*_B_ = 1.00). In HVC (Figure 3B), there was no main effect of sex (*F*_1,25_ = 2.31, *p* = .141, *ω* ^2^ = .04), nor season (*F*_2,25_ = 0.07, *p* = .937, *ω* ^2^ < 0), nor an interaction (*F*_2,25_ = 0.43, *p* = .657, *ω* ^2^ < 0). Descriptive statistics (mean ± SE) for all volume measurements, split by sex and season, are shown in Table 2.

**TABLE 2:** Descriptive statistics (means ± SEs) of Area X and HVC volumes in black‐capped chickadees, split by season, sex, and age.

Structure	Sex	AHY			HY
		Spring	Summer	Winter	Summer
Area X	♂	0.503 ± 0.042 mm^3^	0.465 ± 0.102 mm^3^	0.328 ± 0.033 mm^3^	0.447 ± 0.054 mm^3^
	♀	0.295 ± 0.069 mm^3^	0.246 ± 0.042 mm^3^	0.288 ± 0.063 mm^3^	0.359 ± 0.062 mm^3^
HVC	♂	0.228 ± 0.049 mm^3^	0.270 ± 0.007 mm^3^	0.245 ± 0.052 mm^3^	0.216 ± 0.038 mm^3^
	♀	0.201 ± 0.026 mm^3^	0.150 ± 0.076 mm^3^	0.154 ± 0.035 mm^3^	0.123 ± 0.041 mm^3^

Abbreviations: AHY, after‐hatch‐year; HY, hatch‐year.

### 3.2 Comparison with Phillmore et al.^30^

**FIGURE 4:**
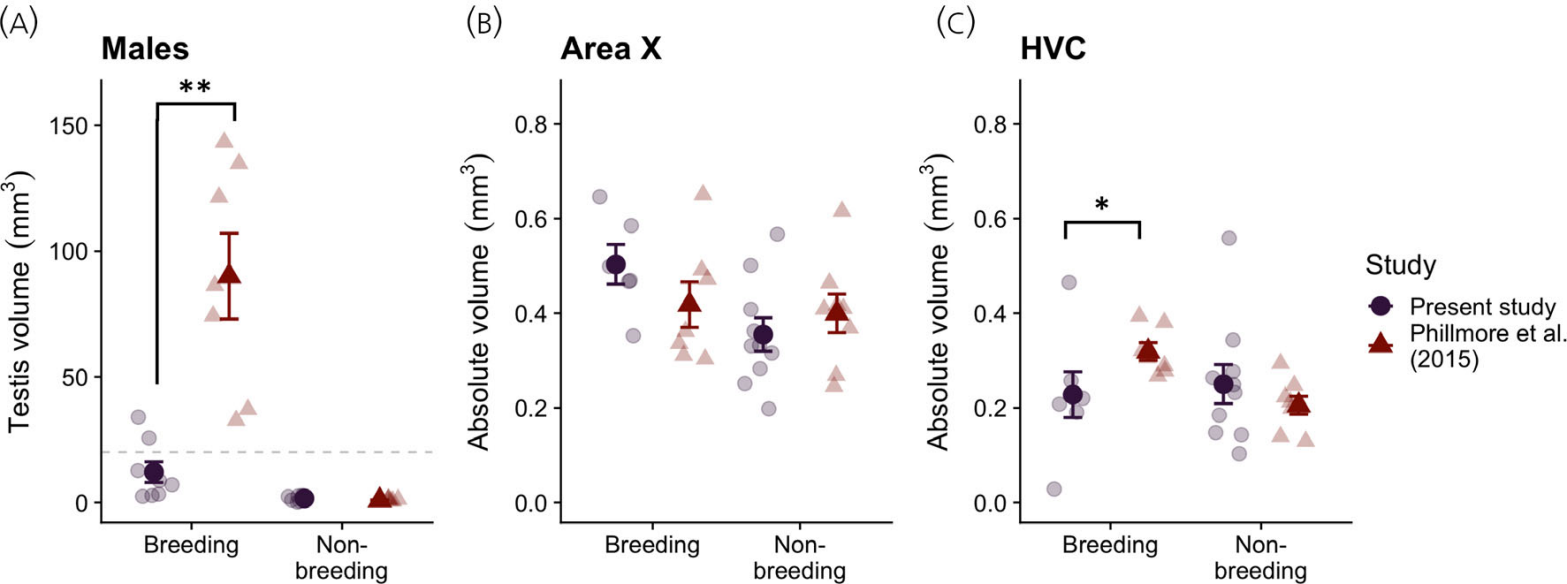
Within‐spring differences in testis volume (A), Area X volume (B) and HVC volume (C) in adult (AHY) male black‐capped chickadees. Asterisk (*) indicates *p* < .05; double asterisk (**) indicates *p* < .001. Dashed line in (A) indicates 20 mm^3^ threshold for breeding condition described by Phillmore et al.^29^ Faded points are individual data; darker points ± error bars indicate group means ± SEs.

We directly compared data from males in the spring group in the present study (*n* = 8) to the breeding males in Phillmore et al.^30^ (*n* = 7) and collapsed the summer and winter males (*n* = 10) in the present study into a non‐breeding group to facilitate comparison with the non‐breeding males in Phillmore et al.^30^ (*n* = 8). We probed for differences in testis volume using a GLM; testis‐volume (natural‐log transformed) was the dependent variable; study and breeding condition were fixed factors. There was a main effect of breeding condition (*F*_1,28_ = 138.1, *p* < .001, *ω* ^2^ = .703) and study (*F*_1,28_ = 11.39, *p* = .002, *ω* ^2^ = .053) on testis volume, and a breeding condition × study interaction (*F*_1,28_ = 25.76, *p* < .001, *ω* ^2^ = .127). We opted to only examine the interaction effect; simple effects tests revealed the breeding males captured in Phillmore et al.^30^ had significantly larger testes compared with spring males in the present study (*t*_28_ = 5.80, *p* < .001), while testis volume did not differ between studies in non‐breeding males (*t*_28_ = −1.24, *p* = .225; Figure 4A). All breeding males captured in Phillmore et al.^30^ had large testes indicative of breeding condition (mean testis volume ± SE for breeding males = 90.02 ± 17.05 mm^3^).

Volume data were not normally distributed when data from both studies were pooled; we therefore used separate Kruskal–Wallis ANOVAs to probe differences between studies in breeding and non‐breeding birds. In both the breeding and non‐breeding groups, Area X did not differ between males in the present study and males captured in Phillmore et al.^30^ (Table 3; Figure 4B). However, breeding males captured in Phillmore et al.^30^ had a significantly larger HVC than spring males in the present study ($\chi_{1}^{2}=5.00$, *p* = .025); HVC volume did not differ between non‐breeding groups ($\chi_{1}^{2}=0.639$, *p* = 0.424), as shown in Figure 4C (also Table 3).

**TABLE 3:** Comparison of Area X and HVC volumes in male black‐capped chickadees between the present study and Phillmore et al.^30^

Structure	Spring/breeding males				Non‐breeding males			
	Present study—early spring	Phillmore et al.^30^—late spring	*χ* ^2^	*p*	Present study	Phillmore et al.^30^	*χ* ^2^	*p*
Area X	0.503 ± 0.042 mm^3^	0.418 ± 0.048 mm^3^	1.31	.253	0.355 ± 0.035 mm^3^	0.400 ± 0.041 mm^3^	1.14	.286
	*n =* 6	*n =* 7			*n =* 10	*n =* 8		
HVC	**0.228 ± 0.049 mm** ^**3**^	**0.319 ± 0.019 mm** ^**3**^	**5.00**	**.025**	0.250 ± 0.041 mm^3^	0.206 ± 0.019 mm^3^	0.639	.424
	*n =* 7	*n =* 7			*n =* 10	*n =* 8		

*Note*: Early spring males captured in the present study between 25 March and 10 April 2021. Late spring males in Phillmore et al.^30^ captured between 15 April and 14 May 2008. Non‐breeding males in the present study consisted of birds captured between 13 August and 12 September 2021 and 1 December 2021 and 26 January 2022; non‐breeding males in Phillmore et al.^30^ captured between 26 January 2008 and 19 February 2009. Data are means ± SEs. Bold text indicates significant difference at *p* < .05.

### 3.3 Age differences (HY and AHY birds)—Summer only

We probed for age differences in our dependent measures (gonads, gonadal steroids, neural measures) only within the group of birds captured in the summer. As we did not intend to capture HY birds in summer, all analyses presented are exploratory in nature. All summer males (both HY and AHY) had testes smaller than 20 mm^3^ (Figure S1A). A Student's *t*‐test revealed no age differences in testis volume (natural‐log‐transformed) in summer males (*t*_8_ = 2.00, *p* = .081). In females, stage of ovary development did not differ by age; all females (HY and AHY) captured in summer had non‐stimulated ovaries (all scores = 1; Figure S1B). We did not compare age differences in circulating gonadal steroids statistically, as we had limited samples in the summer group (Table 1), but individual data are shown in Figure S1C,D. As in adults (described above), volume data from birds captured in summer were analyzed using GLMs, with nucleus volume as the dependent variable, age and sex as the fixed factors, and telencephalon volume as a covariate. There was no main effect of age (*F*_1,11_ = 0.195, *p* = .668, *ω* ^2^ < 0), sex (*F*_1,11_ = 2.07, *p* = .178, *ω* ^2^ = .051), nor an interaction (*F*_1,11_ = 0.579, *p* = .463, *ω* ^2^ < 0) on Area X volume in summer birds (Figure S2A). In HVC, there was similarly no main effect of age (*F*_1,8_ = 3.80, *p* = .073, *ω* ^2^ = .16), sex (*F*_1,8_ = 1.73, *p* = .224, *ω* ^2^ = .04), nor an interaction (*F*_1,8_ = 0.04, *p* = .850, *ω* ^2^ < 0; Figure S2B).

## 4 DISCUSSION

Here, we examined seasonal differences in the volume of vocal control nuclei Area X and HVC in a wild population of male and female black‐capped chickadees. In contrast to some prior studies of seasonal neural plasticity in this species (e.g., Ref. [27]) birds were sacrificed shortly after capture and were not held in any form of short‐ or long‐term laboratory captivity. We quantified two measures of reproductive state, gonad size (testis) or stage (ovaries) and circulating gonadal steroids; to assess how changes in the vocal control system might interact with changes in the HPG axis, we also quantified neural plasticity by measuring the volume of two vocal control structures: Area X (critical for vocal learning) and HVC (critical for vocal production^13^, ^14^).

### 4.1 Seasonal differences in HPG axis activity

Although both AHY males and females in the summer and winter groups (captured 13 August–15 September and 1 December–26 January, respectively) had regressed gonads (testis volume <20 mm^3^; ovary score = 1 or 2) as expected, most AHY birds captured in early spring (from 25 March to 10 April) did not have gonads matching breeding condition: only two of the eight males captured had testes larger than 20 mm^3^, and only one of the eight females had an ovary score ≥3. Similarly, spring birds did not have elevated levels of gonadal steroids: levels of circulating T (males) and E2 (females) did not differ between spring and winter birds (Figure 2C,D).

As this is, to our knowledge, the first study to quantify seasonal differences in circulating gonadal steroids in wild black‐capped chickadees, we cannot compare our data to previously established benchmarks for breeding condition as we can for gonads. But, previous studies of other parid species similarly suggest the birds we captured in early spring were not fully in breeding condition. In willow tits (*Poecile montanus*), a Eurasian tit species closely related to black‐capped chickadees,^45^ plasma T levels in free‐living males rise from about 500 pg/mL in February and March to an April peak of about 1500 pg/mL.^46^ Here, four of the five spring males had plasma T between 500 and 1000 pg/mL, considerably below the 1500 pg/mL breeding peak in willow tits and no different from T levels in winter males. In females, Silverin et al.^46^ report an April E2 peak considerably higher (about 800 pg/mL) than the values reported in our study (all birds <30 pg/mL), another indicator birds captured in spring were not in breeding condition. Taken together, we can conclude that the birds captured in spring showed little to no evidence of HPG axis upregulation and were largely not in breeding condition.

The transition from non‐breeding to breeding condition is a gradual one, only beginning once day length has surpassed a critical threshold. Nicholls et al.^47^ estimate the time between the critical day length threshold for photostimulation and peak reproductive ability to be about 3–4 weeks, although this can vary greatly between species and geographic regions.^48^ Although we do not know for certain what the critical day length is for chickadees in our study area, this 3–4 week difference maps almost perfectly to the 25.5 day difference in median capture dates between the spring group in this study and the breeding group in Phillmore et al.^30^ Breeding male chickadees captured in Phillmore et al.^30^ had significantly larger testes than the males captured in the present study, and all of these breeding males (captured between 15 April and 14 May, i.e., later in the spring) exceeded the 20 mm^3^ threshold for breeding condition. Although we cannot make conclusions about chickadees in breeding condition from the data in this study, we can compare both physiology and neuroanatomy of birds captured in the early (this study) and late spring^30^ to begin to understand the timing of these changes across the pre‐breeding and breeding season.

### 4.2 Seasonal differences in vocal control nuclei

Some have hypothesized that the vernal growth of the vocal control system observed in many species is a direct downstream consequence of HPG axis upregulation, particularly the surge in T (e.g., Refs. [8, 17, 18, 19, 20]). According to this longstanding model, we would therefore expect no differences in the size of vocal control nuclei Area X and HVC compared with non‐breeding birds, as most birds captured in early spring showed virtually no evidence of HPG axis upregulation.

HVC volume was not different between breeding and non‐breeding birds, aligning with the longstanding model. This finding is also similar to the results of Smulders et al.,^28^ but not with Phillmore et al.^29^, ^30^ nor MacDougall‐Shackleton et al.^27^ However, and perhaps unexpectedly, birds captured in the spring had a significantly larger Area X than birds captured in the winter. Before this study, only MacDougall‐Shackleton et al.^27^ observed a difference in Area X between long‐and short‐day, captive black‐capped chickadees. Neither Phillmore et al.^29^ nor Phillmore et al.,^30^ who housed chickadees in short periods of captivity pre‐sacrifice (7 days and overnight, respectively), nor Smulders et al.,^28^ who sacrificed birds immediately after capture, detected differences in Area X. Seasonal differences in Area X were also not detected in a study of wild willow tits and great tits (*Parus major* ^44^).

One explanation of the incongruity among all studies could be captivity: while we and Smulders et al.^28^ minimized time before sacrifice, other studies held birds in the laboratory for varying lengths of time. Our assumption is that data from birds not held in captivity before data collection is more representative of typical seasonal fluctuations in neural plasticity, and that, both length of time of captivity and type of stimulation may alter these typical responses.

Both we and Smulders et al.^28^ did not detect a seasonal difference in HVC; in both studies birds were sacrificed immediately after capture. In the wild, chickadees are vocal throughout the year.^29^, ^49^ The *fee‐bee* song, although most frequently heard in spring, is heard year‐round; other complex learned vocalizations (e.g., *chick‐a‐dee*, *gargle* calls) are similarly produced throughout the year.^49^ Thus, the behavioral demand on HVC, a motor nucleus critical for vocal production, is fairly stable across seasons relative to other species.^25^

Previous studies of birds in captivity could have also reduced the need for vocalizing overall, revealing possible seasonal differences in HVC masked in wild birds. While MacDougall‐Shackleton et al.^36^ state that photostimulated males did sing the *fee‐bee* song, they did not quantify singing in this study. Avey et al.^50^ show chickadees held in captivity vocalize in the typical diurnal and seasonal patterns of wild chickadees, but the amount of vocalizing and singing in captivity does appear to be markedly different from that of the wild: the proportion of *fee‐bee* songs relative to other vocalizations is considerably lower than in the wild, as are *chick‐a‐dee* calls.^49^, ^50^

Captivity may also explain MacDougall‐Shackleton et al.'s^27^ finding in HVC: those chickadees experienced prolonged exposure to constant photoperiods (unlike the gradually changing photoperiods experienced in the wild) and therefore the effects may not be representative of the more subtle effects observed in wild birds.^25^, ^30^ Captivity however does not explain the difference between our results and Smulders et al.^28^ One possibility is that our birds were captured much earlier in spring, however, Area X volume did not differ between early spring males in this study and late spring males in Phillmore et al.,^30^ despite the about 3–4 week difference in capture date between the two studies. Other than timing of capture, our experimental approach is identical to that of Smulders et al.^28^ The lack of seasonal difference in both Area X and HVC in Smulders et al.^28^ can likely be attributed to small group sample size; most groups consisted of fewer than 5 birds (per sex), with some groups (October, February) only consisting of 1 or 2 adults.

How can we explain our findings in Area X? In adult songbirds, Area X, along with dorsolateral thalamic nucleus (DLM) and the lateral magnocellular nucleus of the anterior nidopallium (LMAN) constitutes the anterior forebrain pathway (AFP), the neural substrate for vocal plasticity.^51^ Lesions to the AFP in adult songbirds affect the variability of vocalizations and impair the ability to adapt or modulate vocalizations in response to change (e.g., Refs [52, 53]). Seasonal changes in Area X are observed in adult canaries (*Serinus canaria*; e.g., Ref. [54]) an open‐ended song learner that shows evidence of seasonal vocal plasticity even in adulthood.^14^ Black‐capped chickadees have a wide array of learned vocalizations^55^, ^56^; adults also show evidence of vocal plasticity in the non‐breeding season.^57^ Further, black‐capped chickadees are one of a handful of species who, during breeding season, adjust the frequency of their *fee‐bee* song to match the frequency of nearby conspecifics,^58^, ^59^ a phenomenon known as pitch matching. MacDougall‐Shackleton et al.^27^ suggest that a small, non‐recrudesced HVC in breeding black‐capped chickadees may be sufficient for vocal imitation, but not long bouts of stereotyped song. In the context of pitch matching, it may be that a breeding‐sized HVC is similarly not required, but a significantly larger Area X is, which could explain the significantly larger Area X we observe here even in early spring. The neural correlates of pitch matching in black‐capped chickadees (or other “pitch matching” species) have not been characterized, but given its role in vocal plasticity and sensorimotor integration,^51^, ^60^ Area X is a prime candidate for future investigation.

### 4.3 Regional differences in vernal recrudescence of the vocal control system

The lack of seasonal difference in HVC in the males captured in the present study, compounded by the fact that most males captured in March–April were not in breeding condition, led us to compare our data to the males captured in Phillmore et al.^30^ We show that birds captured in early spring with sub‐breeding levels of T do not have a large HVC by volume, and HVC is significantly smaller when compared with another cohort of birds captured slightly later in the spring with large gonads (and presumably high levels of T). This suggests that the upregulation of the HPG axis, and specifically sufficient levels of T, is at least partially necessary before there can be a complete vernal recrudescence of HVC in this species. In contrast, Area X was fully recrudesced ahead of reproductive maturation, indicating that vernal growth of the vocal control system occurs sequentially, or independently in each region, in black‐capped chickadees. Previous field studies of other songbird species,^21^, ^61^ including other parids,^22^ have similarly shown a complete or partial recrudescence of vocal control nuclei in advance of reproductive maturation, although this is the first study to show a recrudescence of Area X before a recrudescence of HVC.

According to the longstanding model, the reason HVC is the first vocal control nucleus to recrudesce in spring is because of its direct responsiveness to the vernal surge in T: T is a primary effector of HVC growth; HVC expresses relatively large quantities of androgen receptors (ARs) and in contrast, Area X expresses little to no ARs ^11^, ^17^, ^62^(but see Ref [^10^]). In captive white‐crowned sparrows (*Zonotrichia leucophrys gambelii*) held on long photoperiods and given T implants, Tramontin et al.^63^ showed HVC grew to almost maximal size after 7 days, whereas RA and Area X were not significantly larger until after 20 days; this same sequence (i.e., HVC, then X) is also observed in developing zebra finches.^64^ These and other studies (e.g., Refs. [65, 66]) suggest the seasonal effects of T on RA and Area X are not direct, but are mediated indirectly via HVC efferents.^16^ While projection neurons from Area X to HVC do express ARs,^67^ the overall role of T on the HVC to Area X connection is not well‐studied, especially relative to other HVC afferents (e.g., HVC‐RA^68^).

This atypical pattern of vocal control system recrudescence in black‐capped chickadees may be because vernal growth in Area X is entirely independent of HPG axis upregulation in this species, and may instead be regulated by environmental or social cues (described above). In other words, a recrudesced Area X may be required to support increased overall vocal production and vocal plasticity (of both learned songs and calls) earlier in the breeding season than vocal behaviors supported by a recrudesced HVC. However, the mechanisms underlying this atypical pattern are still unknown. One possible mechanism may be that that the HPG axis regulates Area X “upstream” of gonadal recrudescence, perhaps via the actions of the gonadotropins. In rodents, both luteinizing hormone receptor (LHCGR) and follicle stimulating hormone receptor (FSHR) mRNA is found throughout the brain, including in the basal ganglia,^69^ suggesting that Area X, a nucleus of the avian basal ganglia (Person et al., 2008),^70^ may also express gonadotropin receptors. Indeed, Bentz et al.^71^ recently found that LHCGR mRNA is differentially upregulated in the ventromedial telencephalon of breeding adult tree swallows (*Tachycineta bicolor*) during incubation; the role of this gene in the songbird telencephalon is unknown and warrants future study. Another possibility is that even though HVC does not recrudesce first in black‐capped chickadees, HVC may still be promoting (or prioritizing) localized neuronal recruitment to Area X before recrudescing itself, perhaps by the same mechanisms described previously in emberizid sparrows (e.g., via brain‐derived neurotrophic factor, BDNF^16^). We also note that our measure of volume change (or in the case of HVC, lack of volume change) may not be representative of other changes in, for example, neuronal precursors; other histological or immunohistochemical markers of neural plasticity (e.g., proliferating cell nuclear antigen, PCNA; doublecortin, DCX; ^72^, ^73^, ^74^) may be useful in elucidating whether the overall volumetric differences documented here are mirrored by changes in neuronal precursor dynamics.

### 4.4 Sex and age differences in the vocal control system

Sex differences in the volume of Area X have been reported across many songbird species^14^, ^75^ including in studies of wild and captive black‐capped chickadees^27^, ^28^, ^29^, ^30^, ^76^; we replicate this here: males have a larger Area X than females. While this male bias in Area X is believed to parallel the male‐bias in song learning, the role of Area X in supporting vocal learning and production in females also warrants future investigation: despite the overall main effect of sex on Area X volume, of the five birds with the largest Area X, two were female.

We did not find a sex difference in HVC volume, contradicting much of the previous work in black‐capped chickadees (e.g., Refs. [27, 28, 29, 76]). While this may be another product of our spring group not being in breeding condition, interestingly, Phillmore et al.^30^ similarly did not detect a sex difference in HVC volume (as a proportion of telencephalon) in their sample. Phillmore et al.^30^ point to the lack of captivity (only overnight) relative to most other previous studies as a potential explanation for the lack of sex difference as well as small samples (in the context of small HVC effects; described above) which may also explain our findings here.

Sex differences in the vocal control system are proportional to the magnitude of the sex difference in vocal behavior.^75^, ^77^, ^78^ While males and female chickadees differ in their production of the *fee‐bee* song (Odum, 1942),^79^ both sexes produce other learned vocalizations (e.g., *gargle*, *chick‐a‐dee*, *tseet* calls^49^, ^55^, ^80^). Whether there are sex differences in the production of these other, non‐song learned vocalizations is not well understood (but see^27^), however both male and female black‐capped chickadees show evidence of vocal plasticity (specifically in the *chick‐a‐dee* call) during the non‐breeding season.^57^ Furthermore, recent work by Mischler et al.^81^ showed it is actually call production (specifically *gargle* calls), not the *fee‐bee* song, that drives the most activity in HVC and RA in both male and female black‐capped chickadees, suggesting that the role of these nuclei (and perhaps the vocal control system as a whole) is not specific to song, but rather learned vocalizations. The lack of sex difference in HVC reported here might be more reflective of a lack of sex difference in the overall behavioral demand on HVC in chickadees.

Although we originally aimed to only capture adult (AHY) birds, an unexpectedly large number of hatch‐year (HY) birds (captured mostly in August–September) allowed us to explore developmental differences in our neural measures. Juvenile songbirds (including HY birds) are rarely examined in seasonal studies of neural plasticity (especially in wild populations), given that the inherent plasticity associated with development^13^, ^82^ might confound the seasonal plasticity observed in adults. Yet here, we show no difference in the volume of Area X or HVC between HY and AHY black‐capped chickadees captured in August–September. To our knowledge, only Smulders et al.^28^ directly examined developmental differences in the vocal control system of black‐capped chickadees across seasons, finding adult males had a significantly larger HVC than juvenile males, and no age differences in Area X. Given the relatively small number of birds used in the developmental comparisons here, and in Smulders et al.,^28^ we cannot draw strong conclusions from these data, but future work on this topic is warranted to separate the seasonal changes in the vocal control system from those associated with ontogeny.

## 5 CONCLUSIONS

In this study we examined seasonal change in the vocal control system of wild black‐capped chickadees not exposed to any period of laboratory‐based captivity. We quantified volume of vocal control nuclei Area X and HVC using standard histological techniques; while this measure has its drawbacks,^83^ it allows us to easily compare our findings to a considerable body of literature, replicating some previous findings, while contradicting others. We also add to the growing body of work in wild birds showing that the vernal growth of the vocal control system in black‐capped chickadees partially precedes reproductive maturation, challenging previous understanding of the sequence of changes leading to breeding condition. Our results may also be specific to the context of natural behavior in black‐capped chickadees: for example, chickadee‐specific demands on specific vocal control nuclei during the pre‐breeding period. Ultimately, our findings emphasize the importance of ensuring high levels of ecological validity when examining brain–behavior interactions in wild birds to mitigate potentially confounding effects of captivity; and overall, emphasize the important of studying brain‐behavior interactions in non‐traditional songbird models, particularly those with unique life histories and behaviors.

## AUTHOR CONTRIBUTIONS

**Broderick M. B. Parks:** Conceptualization; data curation; formal analysis; investigation; methodology; validation; visualization; writing – original draft; writing – review and editing. **Kyle McVea:** Formal analysis; investigation; validation; writing – review and editing. **Leslie Phillmore:** Conceptualization; funding acquisition; methodology; project administration; resources; supervision; writing – review and editing.

## FUNDING INFORMATION

This work was directly supported by a Discovery Grant (RGPIN‐2018‐04060) awarded to LSP by the Natural Sciences and Engineering Research Council of Canada (NSERC). At the time of this work, BMBP was additionally supported by a NSERC Canada Graduate Scholarship‐Master's (CGS‐M), a Killam Predoctoral Fellowship (Level I) from the Killam Trusts, and a Master's level Nova Scotia Graduate Scholarship (Life Sciences).

## CONFLICTS OF INTEREST STATEMENT

The authors of the manuscript have no conflicts of interest to declare.

## PEER REVIEW

The peer review history for this article is available at https://www.webofscience.com/api/gateway/wos/peer‐review/10.1111/jne.13375.

## Supporting information


**Supplemental Materials.** Supplemental Method and Table S1 ‐ Consistency and reliability of volume measures.

**Figure S1.** Developmental differences in HPG axis activity in black‐capped chickadees captured in the summer group; AHY, after‐hatch‐year; HY, hatch‐year. (A) shows testis volume in males; dashed line indicates 20 mm^3^ threshold for breeding condition described by Phillmore et al.^29^ (B) shows stage of ovary development in females; (C) shows plasma testosterone (T) in males; and (D) shows plasma 17β‐estradiol (E2) in females. Dots are individual data.

**Figure S2.** Developmental differences in vocal control nuclei Area X (A) and HVC (B) in black‐capped chickadees captured in the summer group, separated by sex; AHY, after‐hatch‐year; HY, hatch‐year. Faded points are individual data; darker points ± error bars indicate group means ± SEs.

## Data Availability

Data that support the findings of this study are available at https://doi.org/10.5683/SP3/QBMB5S. This study's analyses also include comparisons to a subset of data from an original study by Phillmore et al.^30^ published in the journal *Developmental Neurobiology* (doi:10.1002/dneu.22220; John Wiley & Sons, Inc.). The data from Phillmore et al.^30^ used in this study's analyses are also available at the above link and are shared under data re‐use rights retained by LSP. Code used for analyses are available from the corresponding author upon reasonable request.
