# Supplementary material for: Vernal growth of vocal control nucleus Area X, but not HVC, precedes gonadal recrudescence in wild black‐capped chickadees (Poecile atricapillus)
Source: J Neuroendocrinol. 2024 Feb 20;37(6):e13375. doi: 10.1111/jne.13375 (PMC12145946; doi:10.1111/jne.13375)
Supplement: Supplementary file 1 — Supplemental Materials. Supplemental Method and Table S1 ‐ Consistency and reliability of volume measures. [file JNE-37-e13375-s003.docx]

**Supplemental Materials**

**Supplemental Method**

**Hormone assays**

We quantified testosterone (T) and 17β-estradiol (E2) in plasma using commercially available enzyme-linked immunosorbent assay (ELISA) kits (T: Enzo Life Sciences, cat no. ADI-900-065; E2: Salimetrics, cat no. 1-3702), following manufacturer instructions. Both ELISA kits have been used extensively to quantify T and E2 in songbird plasma (e.g., Hall and MacDougall-Shackleton, 2012; Wilcoxen et al., 2015; Davies and Sewall, 2016; Berzins et al., 2018). As there was limited plasma per bird, we could only assay T and E2 in a subset (*n*=25) of birds (see Table 1; main article). Plasma samples were diluted to 1:30 for the T assay (e.g., Davies and Sewall, 2016) and 1:20 for the E2 assay (e.g., Berzins et al., 2018). In cases where plasma volume was insufficient (*n*=3 females), the dilution factor for these samples were adjusted accordingly.

T and E2 were generally assayed using trunk blood samples, however due to limited plasma quantities, for some birds (*n=*1 male; *n*=3 females) we instead assayed plasma samples collected in the field. We therefore checked for differences in T and E2 between both sample types (i.e., field vs. trunk) using Wilcoxon’s signed rank tests (similar to Lipshutz and Rosvall, 2021) and found no significant differences (T: *W*=10.0, *p*=0.125; E2: *W*=3.00, *p*=0.500). Samples were assayed in duplicate; intra- and inter-assay variation was <15% for both assays (intra-assay: T=10.7%, E2=4.6%; inter-assay: T=13.5%, E2: 8.4%), consistent with the values reported by the manufacturers (intra-assay: T=10.8%, E2=7.0%; inter-assay: T=14.6%; E2: 6.0%).

**Histology**

One set of tissue was removed from the freezer, washed once in PBS, float mounted serially on 1% gelatinized slides, then air dried for 48 hours. Tissue was then stained for Nissl substance using cresyl violet following a protocol originally described by Ormerod et al. (2003), adapted specifically for avian brain tissue. Briefly, slides were rinsed first in distilled water (to remove salts), then in a 2% cresyl violet acetate (Acros Organics) in distilled water solution for 5-7 minutes. Slides were rinsed again in distilled water, then submerged in glacial acetic acid in 70% ethanol for 30 seconds. We then serially dehydrated slides in two rinses of 95% ethanol and one rinse of 100% ethanol (2 minutes each) before clearing in Neo-Clear (Harleco) for 10 minutes. We then coverslipped slides with Permount (Fisher); slides were then left to dry overnight.

**Consistency and repeatability of volume measurements**

One experimenter (KM) quantified the volume of Area X and HVC in all birds, while another experimenter (BMBP) quantified telencephalon volume. To ensure volume measurements were both consistent between observers and repeatable within observers, we calculated an intraclass correlation coefficient (ICC; reviewed in Liljequist et al., 2019) for all volume measurements using the “Rater Reliability” feature in the seolmatrix module in jamovi (The jamovi project, 2022; Seol, 2023), based on the R package *irr* by Gamer et al. (2019).

For each comparison, we used a one-way ICC model assessing absolute agreement; the unit of analysis was set to “average” as each volume measurement comprised multiple area measurements (Shrout and Fleiss, 1987). Values closer to 1 indicate higher agreement. Between- and within-experimenter ICCs were calculated using volume data collected from subset of birds (*n=*10 for Area X and telencephalon; *n*=9 for HVC) comprising both sexes, both age groups (hatch-year, after-hatch-year), and all capture seasons, as determined by block randomization. Volume measurements for all structures (Area X, HVC, telencephalon) were both highly consistent between observers (ICCs = 0.818-0.991, all *p*s < 0.01) and repeatable within observers (ICCs = 0.967-0.999, all *p*s < 0.001), as shown in Table S1.

**References (Supplemental)**

Berzins LL, Shrimpton JM, Dawson RD (2018) Experimentally altering pre-breeding sex steroids reduces extra-pair paternity in female tree swallows. Ethology 124:410–422.

Davies S, Sewall KB (2016) Agonistic urban birds: elevated territorial aggression of urban song sparrows is individually consistent within a breeding period. Biology Letters 12:20160315.

Hall ZJ, MacDougall-Shackleton SA (2012) Influence of Testosterone Metabolites on Song-Control System Neuroplasticity during Photostimulation in Adult European Starlings (Sturnus vulgaris). PLOS ONE 7:e40060.

Gamer, M, Jim Lemon J, Singh I (2019) *irr: Various Coefficients of Interrater Reliability and Agreement*. [R package]. Retrieved from https://CRAN.R-project.org/package=irr.

Lipshutz SE, Rosvall KA (2021) Nesting strategy shapes territorial aggression but not testosterone: A comparative approach in female and male birds. Hormones and Behavior 133:104995.

Ormerod BK, Lee TT-Y, Galea LAM (2003) Estradiol initially enhances but subsequently suppresses (via adrenal steroids) granule cell proliferation in the dentate gyrus of adult female rats. Journal of Neurobiology 55:247–260.

Seol H (2023) *seolmatrix: Correlations suite for jamovi*. [jamovi module]. Retrieved from https://github.com/hyunsooseol/seolmatrix.

Shrout PE, Fleiss JL (1979) Intraclass correlations: Uses in assessing rater reliability. Psychological Bulletin 86:420–428.

The jamovi project (2022). *jamovi*. [Computer Software]. Retrieved from <https://www.jamovi.org>.

Wilcoxen TE, Horn DJ, Hogan BM, Hubble CN, Huber SJ, Flamm J, Knott M, Lundstrom L, Salik F, Wassenhove SJ, Wrobel ER (2015) Effects of bird-feeding activities on the health of wild birds. Conservation Physiology 3:cov058.

**Supplemental Tables**

**Table S1.** Consistency and reliability of volume measures

| **Comparison** | **Brain region** | **ICC** | **95% CI**  **(lower-upper bounds)** | ***F*** | **Degrees of freedom** | ***p*** |
| --- | --- | --- | --- | --- | --- | --- |
| Inter-scorer consistency | Area X | 0.818 | 0.314-0.954 | 5.51 | 9, 10 | 0.007 |
|  | HVC | 0.952 | 0.802-0.989 | 20.71 | 8, 9 | < 0.001 |
|  | Telencephalon | 0.991 | 0.964-0.998 | 106.22 | 9, 10 | < 0.001 |
| Intra-scorer repeatability | Area X | 0.967 | 0.873-0.992 | 29.86 | 9, 10 | < 0.001 |
|  | HVC | 0.984 | 0.934-0.996 | 61.83 | 8, 9 | < 0.001 |
|  | Telencephalon | 0.999 | 0.994-1.000 | 668.97 | 9, 10 | < 0.001 |

**Note.** ICC = intra-class correlation coefficient; CI = confidence interval
