# Supplementary figures and images for: Vernal growth of vocal control nucleus Area X, but not HVC, precedes gonadal recrudescence in wild black‐capped chickadees (Poecile atricapillus)
Source: J Neuroendocrinol. 2024 Feb 20;37(6):e13375. doi: 10.1111/jne.13375 (PMC12145946; doi:10.1111/jne.13375)

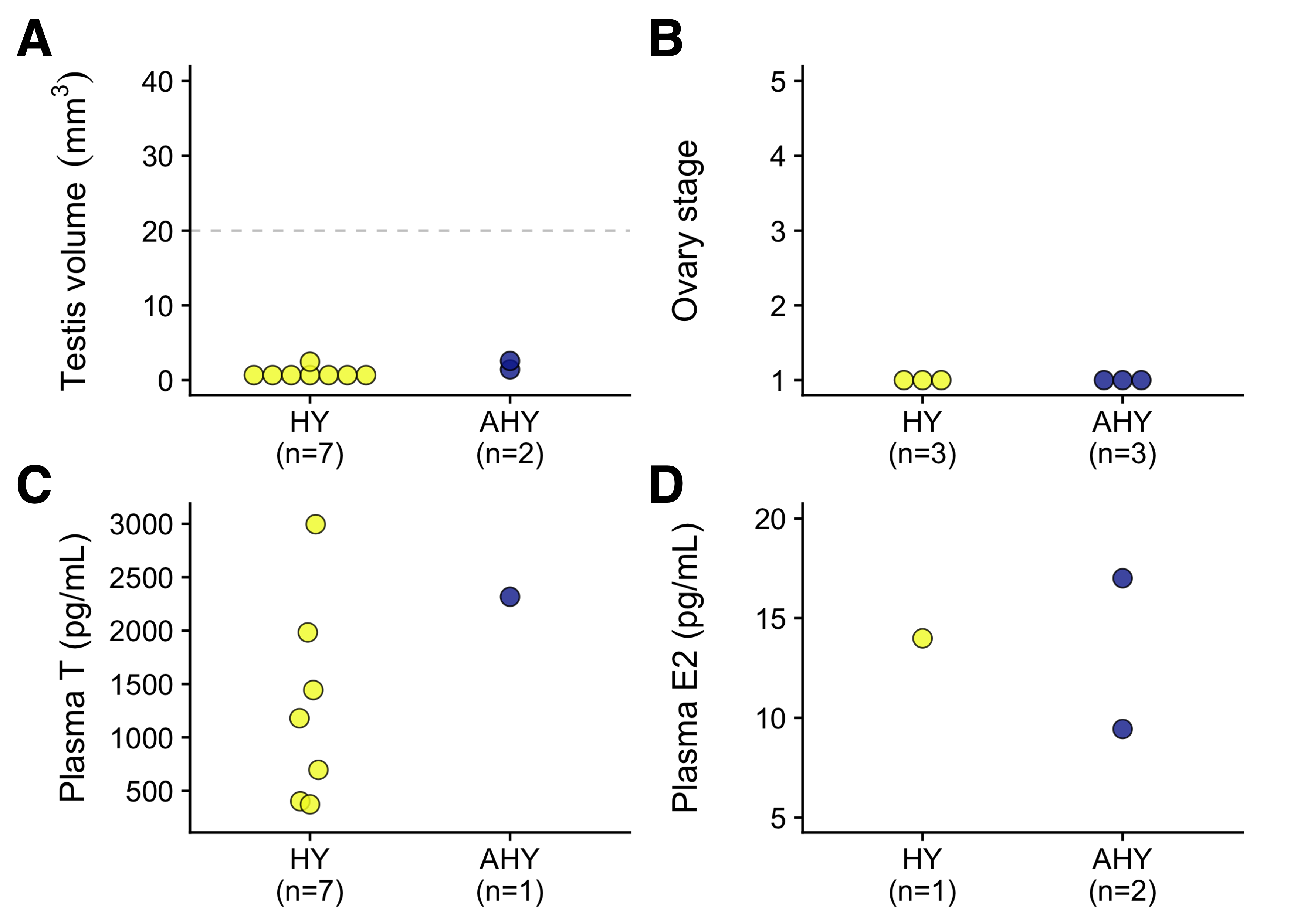

Supplement: Supplementary file 2 — Figure S1. Developmental differences in HPG axis activity in black‐capped chickadees captured in the summer group; AHY, after‐hatch‐year; HY, hatch‐year. (A) shows testis volume in males; dashed line indicates 20 mm3 threshold for breeding condition described by Phillmore et al. 29 (B) shows stage of ovary development in females; (C) shows plasma testosterone (T) in males; and (D) shows plasma 17β‐estradiol (E2) in females. Dots are individual data. [file JNE-37-e13375-s001.png]

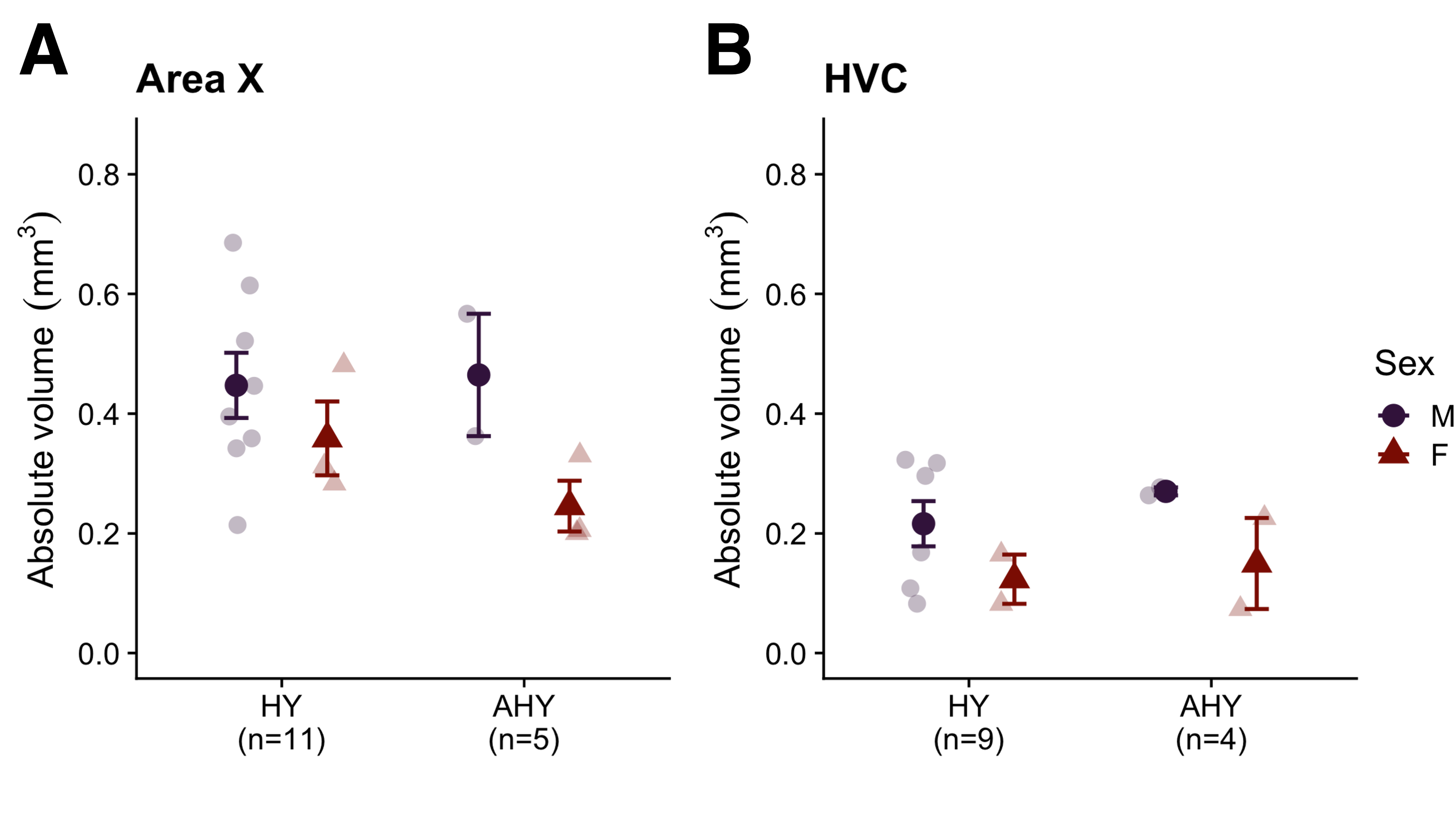

Supplement: Supplementary file 3 — Figure S2. Developmental differences in vocal control nuclei Area X (A) and HVC (B) in black‐capped chickadees captured in the summer group, separated by sex; AHY, after‐hatch‐year; HY, hatch‐year. Faded points are individual data; darker points ± error bars indicate group means ± SEs. [file JNE-37-e13375-s002.png]
